# What Imaging Modality Is More Effective in Predicting Early Recurrence of Hepatocellular Carcinoma after Hepatectomy Using Radiomics Analysis: CT or MRI or Both?

**DOI:** 10.3390/diagnostics13122012

**Published:** 2023-06-09

**Authors:** Qing Wang, Ye Sheng, Zhenxing Jiang, Haifeng Liu, Haitao Lu, Wei Xing

**Affiliations:** 1 Department of Radiology, Third Affiliated Hospital of Soochow University, Changzhou First People’s Hospital, Changzhou 213200, China; wangqing1104@outlook.com (Q.W.); jzparker@sina.com (Z.J.); liuhfyx@163.com (H.L.); vluhaitao@163.com (H.L.); 2 Department of Interventional Radiology, Third Affiliated Hospital of Soochow University, Changzhou First People’s Hospital, Changzhou 213200, China; airjordansy@outlook.com

**Keywords:** hepatocellular carcinoma, early recurrence, radiomics, prediction

## Abstract

Background: It is of great importance to predict the early recurrence (ER) of hepatocellular carcinoma (HCC) after hepatectomy using preoperative imaging modalities. Nevertheless, no comparative studies have been conducted to determine which modality, CT or MRI with radiomics analysis, is more effective. Methods: We retrospectively enrolled 119 HCC patients who underwent preoperative CT and MRI. A total of 3776 CT features and 4720 MRI features were extracted from the whole tumor. The minimum redundancy and maximum relevance algorithm (MRMR) and least absolute shrinkage and selection operator (LASSO) regression were applied for feature selection, then support vector machines (SVMs) were applied for model construction. Multivariable logistic regression analysis was employed to construct combined models that integrate clinical–radiological–pathological (CRP) traits and radscore. Receiver operating characteristic (ROC) curves, calibration curves, and decision curve analysis (DCA) were used to compare the efficacy of CT, MRI, and CT and MRI models in the test cohort. Results: The CT model and MRI model showed no significant difference in the prediction of ER in HCC patients (*p =* 0.911). Radiomics^CT&MRI^ demonstrated a superior predictive performance than either Radiomics^CT^ or Radiomics^MRI^ alone (*p* = 0.032, 0.039). The combined CT and MRI model can significantly stratify patients at high risk of ER (area under the curve (AUC) of 0.951 in the training set and 0.955 in the test set) than the CT model (AUC of 0.894 and 0.784) and the MRI model (AUC of 0.856 and 0.787). DCA demonstrated that the CT and MRI model provided a greater net benefit than the models without radiomics analysis. Conclusions: No significant difference was found in predicting the ER of HCC between CT models and MRI models. However, the multimodal radiomics model derived from CT and MRI can significantly improve the prediction of ER in HCC patients after resection.

## 1. Introduction

Hepatocellular carcinoma (HCC) is the fifth most common malignant tumor and the third leading cause of cancer-related death worldwide [1]. Among various available treatments for HCC, hepatectomy remains the first-line curative therapy [2]. Unfortunately, it has been observed that early recurrence (ER) after hepatectomy, within two years, is more prevalent than late recurrence, accounting for over 70% of cases [3,4]. This is caused by the dissemination of residual tumor cells, leading to a much shorter overall survival of 16.2 months in comparison to 65.4 months of late recurrence [5]. Therefore, the identification of individuals with a high risk of ER of HCC preoperatively and the implementation of preventive measures are the most effective methods to ensure cost efficiency and optimize the management of perioperative HCC [6].

Recently, numerous radiomics analyses have been conducted to predict the ER of HCC after hepatectomy [7,8,9]. Most radiomics studies derived from computed tomography (CT) [10,11,12,13,14,15] and magnetic resonance imaging (MRI) with tumor features [16,17,18,19] and peritumoral features [20,21] yielded satisfactory efficacy with AUCs of 0.742–0.873. However, it is still uncertain which modality (CT or MRI) is superior for the prediction of ER in HCC patients after hepatectomy. In previous studies, it was widely recognized that MRI is more effective than CT in detecting and discriminating HCC, especially for small HCC [22,23], and contrast-enhanced MRI (CE-MRI) provides better visualization of HCC characteristics, including capsule enhancement [24]. However, at the pixel and feature levels, a study comparing CT- and MRI-based radiomics analysis for predicting MVI in solitary HCC showed similar predictive performance [25]. Moreover, Ying He et al. demonstrated that a multimodal radiomics model (incorporating CT and MRI) offered more accurate prognostic prediction after hepatectomy than either CT or MRI alone [26].

To our knowledge, there has still been no comparative study as to whether a single-modal radiomics model (CT alone or MRI alone) or a multimodal radiomics model (combined CT and MRI) is more effective in predicting the risk of ER after hepatectomy for HCC. Therefore, we integrated the radscore derived from CT, MRI, and combined CT and MRI, with or without clinical–radiological–pathological traits, and compared their efficacy for predicting the ER risk after HCC hepatectomy.

## 2. Materials and Methods

### 2.1. Patients

**Figure 1:**
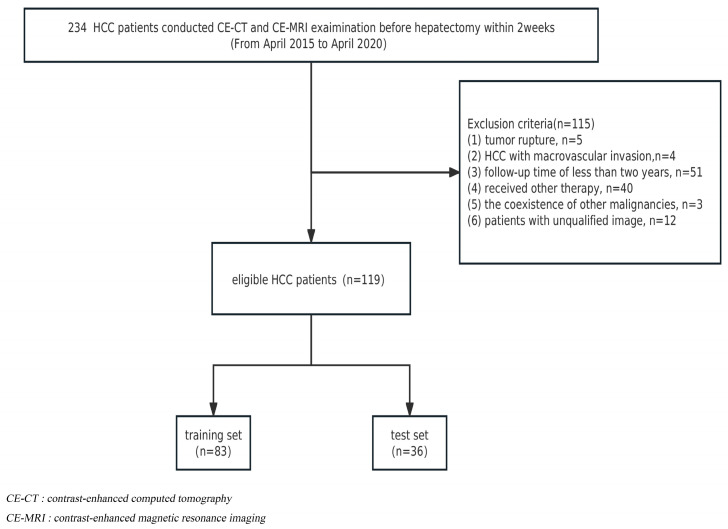
Flowchart of patient selection.

The institutional review board approved this retrospective study, and informed consent was waived. From April 2015 to April 2020, a total of 234 HCC patients receiving radical liver segmentectomy were recruited. The inclusion criteria were as follows: (1) histopathologically diagnosed HCC and (2) contrast-enhanced CT and abdominal MRI examination conducted within 2 weeks before hepatectomy. Exclusion criteria included the following: (1) tumor rupture; (2) HCC with macrovascular invasion (main portal vein or hepatic veins); (3) a follow-up period for patients of less than two years; (4) patients who received radiotherapy, chemotherapy, interventional therapy, or comprehensive therapy before postoperative recurrence; (5) the coexistence of other malignancies; and (6) patients with unqualified images. Finally, 119 patients were included, and the detailed flow chart of the patient selection is presented in Figure 1. At a ratio of 7:3, HCC patients were randomly assigned to either the training cohort (*n* = 83) or the validation cohort (*n* = 36).

### 2.2. Follow-Up and Study Endpoint

All HCC patients were regularly monitored for potential recurrence via CE-CT or CE-MRI at three-month intervals for the initial two years following surgical resection and subsequently at six-month intervals. ER is defined as the presence of new intrahepatic lesions with typical imaging features of HCC or confirmed extrahepatic metastasis by histopathology or tumor staining through TACE within two years post-resection. The initial recurrence was perceived as the endpoint, while the follow-up period terminated in April 2020.

### 2.3. Imaging Techniques

All MR scans were performed with 3.0 T MR scanners (Verio, Siemens Healthcare, Erlangen, Germany). Patients underwent upper abdomen MRI. The comprehensive mpMRI protocol included breath-hold fat-suppressed T2-weighted imaging (T2WI), breath-hold T1-weighted imaging, breath-hold three-dimensional volumetric-interpolated fat-suppressed arterial phase (AP, 20–30 s), portal venous phase (PVP, 60–70 s), and delay phase (DP, 180 s). Contrast T1-weighted images were acquired using gadolinium-diethylenetriamine pentaacetic acid (Gd-DTPA) at a rate of 2.5 mL/s and 0.1 mmol/kg. Unfortunately, the DWI sequence was excluded from the analysis due to inconsistencies in the scanning protocol with varying b-values.

Similarly, CE-CT was conducted with one of the following multidetector row CT (MDCT) scanners: 320-detector row CT (Aquilion One, Toshiba Medical System, Tokyo, Japan; 64-slice LightSpeed VCT (Discovery HD750, GE Healthcare), or Brilliance iCT 256 (Philips Healthcare, Cleveland, OH, USA). Following an unenhanced scan, we administered Ultravist 370, Bayer Schering Pharma, Berlin, Germany, into an antecubital vein at a rate of 3.0 to 3.5 mL/s and 1.5 mL/kg via a pump injector. Precontrast, hepatic arterial, portal venous, and delay phase CT images were obtained at 0 s, 30 s, 60 s, and 180 s with 3 mm reconstructed section thicknesses and 3 mm reconstruction intervals.

### 2.4. Image Analysis

The qualitative CT/MRI analysis was performed by two abdominal radiologists with 8–10 years of experience in liver imaging (reader 1, Q.W.; reader 2, Y.S.). The observers were apprised of the HCC patient, yet they were blinded to the clinical and pathologic data. In the event of disagreements, the readers engaged in a discussion until a definitive consensus was reached. Intraobserver and interobserver agreement tests were conducted for all traits using Cohen’s kappa statistics.

Radiologic traits assessed from CT and MRI included tumor size, tumor number, wash-in, wash-out, pseudocapsule, necrosis, infiltrative margin, LI-RADS, and liver cirrhosis. Discrepancies in tumor number, wash-in, wash-out, pseudocapsule, necrosis, and the infiltrative margin between CT and MRI were recorded. Tumor size was the average of the tumor diameters derived from the preoperative CT and MRI reports. LI-RADS and liver cirrhosis were evaluated according to CT and MRI. The following clinical and pathologic traits were also analyzed: (a) age; (b) sex; (c) alpha-fetoprotein (AFP); (d) etiology including hepatitis B or C virus (HBV, HCV) and other; (e) CNLC; (f) BCLC; (g) microvascular infiltration; (h) MVI; (i) satellite nodules; and (j) differentiation grade.

### 2.5. Radiomics Analysis

**Figure 2:**
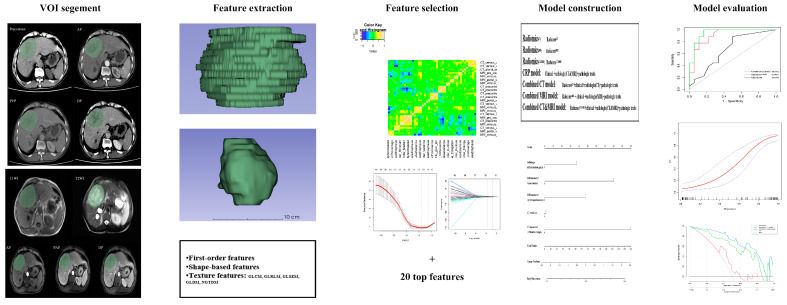
The workflow to conduct radiomics analysis in our study. Tumors were contoured manually and adjusted slice by slice on five MRI sequences and four CT phases. VOI of the whole tumor was segmented, and radiomics features were separately extracted. Maximum relevance minimum redundancy (MRMR) and least absolute shrinkage and selection operator (LASSO) logistic regression were used for feature selection, and SVM was used for radiomics model construction. Receiver operating characteristic curve (ROC), calibration, and decision curve analysis (DCA) were applied for model performance assessment.

The workflow of radiomics analysis is illustrated in Figure 2. Two radiologists with vast experience in liver imaging, reader 1 (Q.W.) and reader 2 (Y.S.), manually delineated the volumes of interest (VOIs) based on the whole tumor on each transverse slice for five MRI sequences (T2WI, T1WI, AP, PVP, DP) and four CT phases (precontrast, arterial, venous, delay) using ITK-SNAP (http://www.itksnap.org/pmwiki/pmwiki.php accessed on 12 April 2023). To ensure minimal bias, a trained radiologist with over a decade of expertise in abdominal imaging, reader 3 (HF.L.), meticulously verified the VOIs.

To eliminate the difference between machines and scanning, the standardized isometric voxels for CT and MRI and masks were set to a size of 1 mm × 1 mm × 1 mm. A total of 3776 CT features and 4720 MRI features per patient were extracted, including 36 first-order features, 14 shape-based features, 150 texture features, and 744 wavelet transformation features.

These feature values were standardized with Z-scores. Redundant features were excluded, and only features with excellent Pearson correlation coefficients (PCC ≥ 0.90) were included in subsequent analyses. Using the MRMR [27] and LASSO regression, the top-ranked twenty valuable features were identified. Subsequently, SVM was used for radiomics model construction with five-fold cross-validation. The radiomics score (radscore) was calculated based on a linear combination of the selected radiomics features multiplied by their respective coefficients for distinguishing between the ER group and the non-ER group. Finally, Radiomics^CT^, Radiomics^MRI^, and Radiomics^CT&MRI^ were established and validated in the test cohort.

Radiomics analysis was carried out with FeAture Explorer Pro (FAE, version. 0.5.2) in Python based on the Pyradiomics package (version: 3.7.6).

### 2.6. Statistical Analysis

Univariate analysis (*p* < 0.1) and the multivariate logistic regression analysis using the forward stepwise method were employed to select independent variables. To discriminate the ER group from the non-ER group, combined models incorporating radscores based on CT, MRI, or CT and MRI features, along with independent clinical–radiological–pathological (CRP) traits, were developed.

The interobserver reproducibility of radiomic features was evaluated using the intraclass correlation coefficient (ICC), with a value greater than 0.75 indicating good reproducibility. The performance of Radiomics^CT^, Radiomics^MRI^, and Radiomics^CT&MRI^ for discriminating the ER group was compared using ROC analysis in the training and test cohorts, with Delong’s test employed for statistical analysis. Furthermore, the proposed predictive models were evaluated for calibration and clinical utility using a calibration curve and decision curve analysis (DCA).

All statistical analyses were conducted by EmpowerStats software (version 4.0, http://www.empowerstats.com accessed on 12 April 2023, X&Y Solutions, Inc., Boston, MA, USA) with R packages (version 4.2.0). Statistical significance was set at *p* < 0.05.

## 3. Results

### 3.1. Patient Information

For all 119 enrolled patients, non-ER was found in 59 patients (41 in the training cohort and 18 in the validation cohort), and ER was found in 60 patients (42 in the training cohort and 18 in the validation cohort), with 46 intrahepatic recurrences, 8 tumor thrombi, and 6 distant metastatic tumors. The baseline characteristics of all patients are listed in Table 1.

### 3.2. Radiologic Characteristics

**Figure 3:**
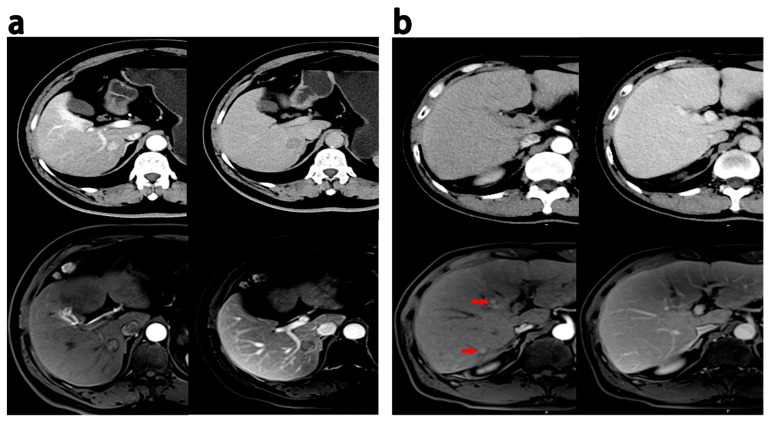
Discrepant MRI and CT findings in HCCs. (**a**) HCC patients (recurrence within 18 months after hepatectomy), 60 years, a 1.9 cm HCC; enhancement in the pseudocapsule was observed in the AP, and delayed phase on MRI while the appearance was not observed on CT. (**b**) HCC patients (ER group), male, 52 years old, 0.6–0.8 cm HCC lesions with wash-in in the AP (red arrow) and partial wash-out in the PVP at MRI; these lesion did not show wash-in and wash-out at CT. PVP = portal venous phase; AP = arterial phase.

All radiologic traits had an excellent intraobserver and interobserver agreement (kappa > 0.75). Intratumoral necrosis and smooth margins were more frequent on MRI than on CT (*p* < 0.05). Nine cases showed multilesions, eleven cases displayed pseudocapsules, five cases revealed intratumoral necrosis, six cases demonstrated infiltrative margins, two cases exhibited wash-in, and thirteen cases presented wash-out on MRI but not on CT. Conversely, eleven cases exhibited multiple lesions, twelve had pseudocapsules, eleven had intratumoral necrosis, seventeen had infiltrative margins, five had wash-in, and eight had wash-out on CT scans but not on MRI. Figure 3 provides representative images of discrepant MR imaging and CT findings.

### 3.3. Radiomics Feature Selection

Among the total CT and/or MRI features, 953 CT radiomics features (25.2%) and 1346 MRI radiomics features (28.5%) showed PCC > 0.90. The top 20 radiomics features ranked in the training dataset were selected. Eventually, Radiomics^CT^ based on seven CT features, Radiomics^MRI^ based on three MRI features, and Radiomics^CT&MRI^ based on two CT features and five MRI features were built. The equation of Radiomics^CT&MRI^ was as follows:

$$\begin{array}{ll} Radscore^{\mathrm{CT}\&\mathrm{MRI}}= & 0.885\times {\mathrm{CT}}_{\mathrm{AP}\_\mathrm{original}\text{\_}\mathrm{firstorder}\text{\_}\mathrm{Median}}-0.701\times {\mathrm{CT}}_{\mathrm{PVP}\text{\_}\mathrm{wavelet}-\mathrm{HHH}\text{\_}\mathrm{gldm}\text{\_}\mathrm{DependenceVariance}}\\ & +0.376\times {\mathrm{MRI}}_{\mathrm{AP}\text{\_}\mathrm{wavelet}-\mathrm{HLL}\text{\_}\mathrm{firstorder}\text{\_}\mathrm{Skewness}}\\ & +0.947\times {\mathrm{MRI}}_{\mathrm{PVP}\text{\_}\mathrm{wavelet}-\mathrm{LHH}\text{\_}\mathrm{glrlm}\text{\_}\mathrm{RunPercentage}}\\ & +0.626\times {\mathrm{MRI}}_{\mathrm{PVP}\text{\_}\mathrm{wavelet}-\mathrm{HL}\text{\_}\mathrm{firstorder}\text{\_}\mathrm{Mean}}\\ & +0.992\times {\mathrm{MRI}}_{\mathrm{PVP}\text{\_}\mathrm{wavelet}-\mathrm{LHL}\text{\_}\mathrm{glrlm}\text{\_}\mathrm{ShortRun}\;\mathrm{HighGrayLevelEmphasis}}\\ & +0.633\times {\mathrm{MRI}}_{\mathrm{DP}\text{\_}\mathrm{original}\text{\_}\mathrm{firstorder}\text{\_}\mathrm{Minimum}} \end{array}$$

In the training cohort, *Radscore^CT&MRI^* of ER patients were generally higher than those of patients without ER (0.75 ± 0.24 vs. 0.23 ± 0.21; *p* < 0.001). The test cohort also confirmed this result (0.75 ± 0.23 vs. 0.34 ± 0.26; *p* < 0.001).

The interobserver reproducibility of the radiomic feature extraction in the Radiomics^CT^, Radiomics^MRI^, and Radiomics^CT&MRI^ models showed high ICC values (median values of 0.941, 0.925, and 0.939, respectively, and ranges of 0.910–0.961, 0.887–0.951, and 0.907–0.960, respectively).

### 3.4. Comparison Performance of Different Models for Discriminating ER vs. Non-ER

**Figure 4:**
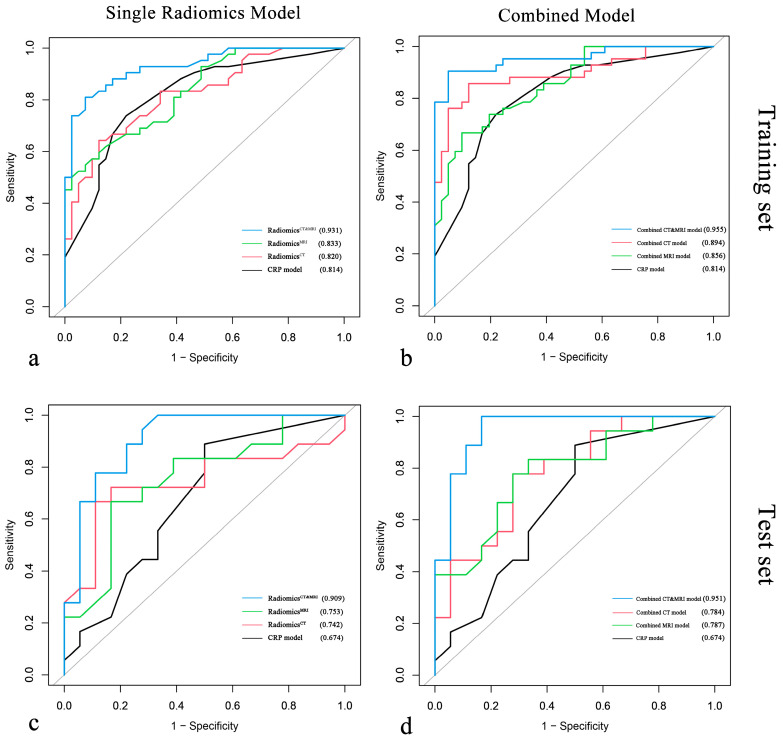
Comparison of ROC curves for single radiomics models vs. clinical–radiological–pathological (CRP) model and for combined models vs. CRP model in the training cohort (**a**,**b**) and the test cohort (**c**,**d**).

Multivariate logistic regression demonstrated that pathologic differentiation grade, tumor number, and intratumoral necrosis measured with MRI and infiltrative margin and wash-out measured with CT were significantly associated with ER of HCC after resection (*p* < 0.050) (Table 2). The performance of all models is summarized in Table 3, and the ROC curve is shown in Figure 4.

**Figure 5:**
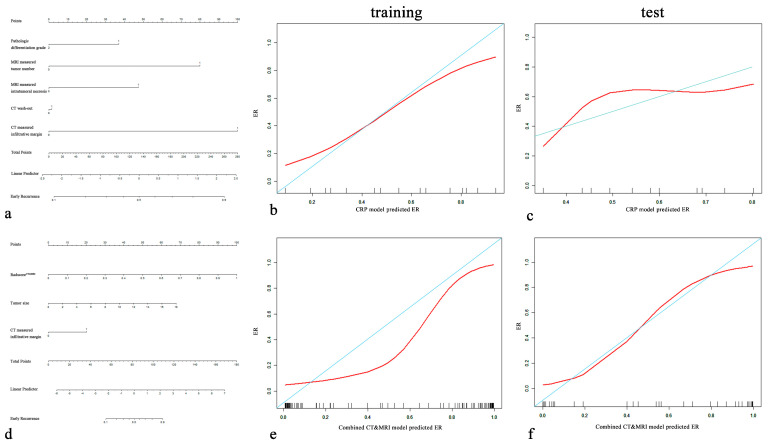
CRP nomogram (**a**) and combined CT and MRI nomogram (**d**). Calibration curves for the CRP nomogram (**b**,**c**) and combined CT and MRI nomogram (**e**,**f**) for the training cohort and test cohort. On the *y*-axis is the actual outcome of early recurrence of HCC, and on the *x*-axis is the predicted outcome. The perfect performance of the ideal model is represented by the blue lines. The red lines represent the performance of the model, of which a closer fit to the blue line indicates better prediction performance.

We compared the performance of radiomics models (Radiomics^CT^, Radiomics^MRI^, Radiomics^CT&MRI^) in the test set, and the AUCs of Radiomics^CT^, Radiomics^MRI^, and Radiomics^CT&MRI^ were 0.742, 0.753, and 0.909, respectively. There was no significant difference between Radiomics^CT^ and Radiomics^MRI^ (*p =* 0.911), while Radiomics^CT&MRI^ showed better significant predictive performance than Radiomics^CT^ (*p* = 0.032) and Radiomics^MRI^ (*p* = 0.039). Meanwhile, Radiomics^CT&MRI^ showed improvement over the CRP model (Figure 5a) in the training set (0.931 vs. 0.814 for AUC, *p* = 0.041) and in the test set (0.909 vs. 0.674 for AUC, *p* = 0.041) for predicting ER risk.

After incorporating independent CRP factors, we compared the performance of the combined models (combined CT model, combined MRI model, and combined CT and MRI model). No significant difference between the combined CT model and the combined MRI model (*p =* 0.464, 0.983). The combined CT and MRI model (Figure 5d) outperformed the combined CT model (*p* = 0.044, 0.010 in the training cohort and test cohort) and combined MRI model (*p* = 0.001, 0.024 in the training cohort and test cohort). The formula of the combined CT and MRI model was as follows:

$$\mathrm{Logit}\left(Y\right)=-11.93+0.60\times \mathrm{tumor}\;\mathrm{size}+2.29\times {\mathrm{CT}}_{\mathrm{infiltrative}\;\mathrm{margin}}+12.88\times {\;\mathrm{radscore}}^{\mathrm{CT}\&\mathrm{MRI}}$$

The combined CT and MRI model and Radiomics^CT&MRI^ in the training set (*p* = 0.083) and the test set (*p* = 0.434) showed no significant difference in predicting the ER risk of HCC. Calibration plots graphically showed agreement between the model-predicted probability of ER and histopathologic ER (Figure 5b,c,e,f).

**Figure 6:**
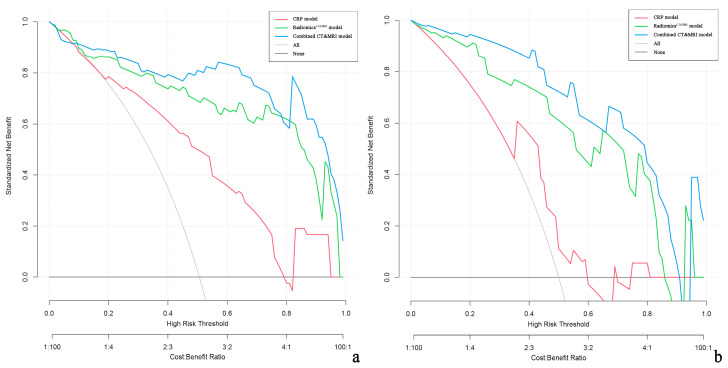
Decision curve analysis (DCA) of the clinical usefulness assessment of the Radiomics^CT&MRI^ model, CRP model, and combined CT and MRI model in the training cohort (**a**) and the test cohort (**b**). The *y*-axis represents the net benefit, and the *x*-axis represents the threshold probability. The combined CT and MRI (blue line) achieves more net benefit than the Radiomics^CT&MRI^ model (green line), CRP model (red line), treat-all strategy (gray line), and treat-none strategy (horizontal black line) across the majority of the range of threshold probabilities.

Decision curve analysis demonstrated that the combined CT and MRI model and Radiomics^CT&MRI^ provided a greater net benefit than CRP models for discriminating high-risk ER patients in both the training and test cohorts (Figure 6).

## 4. Discussion

In this study, we developed and validated radiomics models based on CT, MRI, and CT and MRI. The MRI-based models showed comparable performance to the CT-based models, while the multimodality (CT and MRI) model demonstrated a relative improvement in predicting ER compared to single-modality (CT or MRI alone). Therefore, combining preoperative CT and MRI using radiomics analysis is essential for accurately predicting ER in HCC patients, which aids in the decision-making process for operative treatment and postoperative management.

Radiomics analysis is a cost-effective way to objectively and quantitatively measure intratumor heterogeneity [9]. The results of our study are in agreement with those of previous studies [10,11,12,15,17,20,28], which suggested that CT or MRI using radiomics analysis may be advantageous for predicting ER in HCC after resection. Nonetheless, our study did not detect any distinction between MRI models and CT models (Radiomics^CT^ vs. Radiomics^MRI^, combined CT vs. combined MRI model) for predicting ER in HCC patients. This could be explained by the fact that similar tumor-related information was shown from CT or MRI images. For example, the features of contrast CT and MRI are both mainly concerned with tumor hemodynamics [29]. The traits of T2WI inhomogeneity, which is related to tumor heterogeneity characterized by histological variations and potential rapid growth, are also observed in non-contrast CT scans [30,31]. Thus, there are good reasons to believe that CT or MRI using radiomics analysis might convey similar predictive information and play a similar role in ER prediction after hepatectomy. However, Zhen Zhang et al. suggested that MRI provided better performance for predicting ER of HCC after resection compared with previous CT studies [19]. Their comparative results between different MRI or CT studies limited the reliability. Our included patients who underwent both CT and MRI scans enhanced the reliability of our comparative analyses, and this consistency was explained.

For diagnosing HCC ≤ 3.0 cm, Lee et al. found that multimodal CT and MRI were more sensitive than CT or MRI alone [32]. Similarly, our results at the pixel and feature levels confirmed that Radiomics^CT&MRI^ achieved better performance and higher sensitivity and specificity than Radiomics^CT^ and Radiomics^MRI^ in the training and test cohorts, suggesting that it is imperative and superior to conduct both CT and MRI to predict ER in patients with HCC. The advantage of our combined CT and MRI model compared to the CT or MRI model further confirmed the value of multimodality to predict ER in HCC patients after hepatectomy. Meanwhile, this combined CT and MRI model is comparable to Radiomics^CT&MRI^, showing that CT and MRI may be sufficient for predicting ER, even without clinical, radiological, or pathological traits. A recent study compared multimodal prognostic models for HCC patients and suggested that multimodal radiomics models (CT and MRI) could enhance prognosis prediction accuracy in HCC patients [26]. Therefore, further large-scale studies should be conducted to validate the benefits of CT and MRI.

Investigations have shown that the independent risk factors for predicting ER in hepatocellular carcinoma (HCC) patients are not always consistent. This might be attributed to the presence of selection bias due to stringent inclusion criteria or the heterogeneity of the study populations with various clinicopathological characteristics [5,33]. Our study revealed that pathologic differentiation grade, number of tumors, and intratumoral necrosis measured through MRI, and infiltrative margin and wash-out measured through CT scan were independent risk factors for ER in the CRP model. By merging independent traits with radioscores, the combined model could be a novel and noninvasive way to predict ER in HCC [13,21,34]. In our multimodal model, tumor size and infiltrative margin measured through CT and radscore were risk factors for predicting ER in HCC.

Low differentiation with marked cellular pleomorphism typically represents the worse biological behavior of tumors for HCC [35]. It is line to previous research [17], our study also argued that pathologic differentiation grade was a risk factor for ER in HCC patients after hepatectomy. However, when radiomics signatures were incorporated into the combined models, pathologic differentiation grade was no longer necessary. This may be due to the lower impact on the model than the radscore [10].

MRI showed high sensitivity to small lesions [36] and intratumor necrosis [37]. Our research demonstrated that those with multiple tumors and intratumor necrosis in the MRI scans were more prone to experiencing ER. Multiple tumors with multicentric origin indicated an increased risk of aggressive tumor behavior and metastatic potential [38]. Research has demonstrated that individuals with multiple HCC tumors are more likely to experience ER [13]. A large tumor size or hypoxia may cause intratumor necrosis, which is related to inflammation produced by various host-related conditions [39]. Intratumor necrosis was identified as a sign of predicting treatment efficacy [31,40]. In our research, intratumor necrosis was also found to be a predictor of ER in HCC after resection, reflecting rapid growth and much more aggressive biological behavior [40].

Infiltrative tumor margin measured in CT refers to the invasiveness to peritumoral tissue [19]. Additionally, the presence of wash-out is critical to the diagnosis of HCC [41] and the growth of a malignant tumor [42], which highlights the hemodynamic effects of angiogenesis. In our study, infiltrative tumor margin and wash-out measured through CT were closely correlated with ER in HCC patients, which was partly in line with a previous study [14,43].

Furthermore, the findings of our study indicated that tumor size was a significant risk factor in our multimodal models. Hong et al. [44] even argued that tumor size was the sole significant prognostic factor for ER in HCC patients. This could be because larger tumors are more likely to have a high rate of MVI [45] and a narrower surgical margin during partial hepatectomy [46], which often results in poor recurrence-free survival (RFS).

Decision curve analysis showed that our combined CT and MRI model or Radiomics^CT&MRI^ demonstrated great potential clinical application in predicting ER in HCC. Patients can be risk-stratified according to nomogram scores to advise the period of follow-up. Preoperative TACE or systemic therapy may benefit HCC patients who have a high risk of ER, especially recurrence within 6 months [5]. Furthermore, even for postoperative HCC patients, closer follow-up or postoperative adjuvant therapy is also required to prevent recurrence in high-risk patients [6,47]. Our study provides a practical preoperative individualized prediction of ER and may distinguish patients with HCC who are unsuitable for initial hepatectomy.

There were still several limitations to this study. First, this is a small sample, retrospective single-center study, which may limit the reliability and generalization to other institutions. Second, we only included HCC patients who were injected with Gd-DTPA in our institution, but Zhen Zhang et al. [19] argued that more than 92.3% of early HCC recurrence patients could be correctly identified with the radscore based on Gd-EOB-DTPA-enhanced MR images. Thus, further radiomics analysis based on Gd-EOB-DTPA is still required to confirm the potential advantage between different MRI contrast agents. Third, later recurrence and RFS were not evaluated due to the limited follow-up period of less than 5 years. In addition, manual identification of whole tumors and segmentation of VOIs in CT and MR images are laborious and time-consuming. Deep learning or machine learning should be evaluated for predicting HCC outcomes in future studies [48,49,50,51].

## 5. Conclusions

Radiomics analysis did not demonstrate a significant difference between MRI and CT for predicting ER after hepatectomy. Nevertheless, a multimodal model incorporating both CT and MRI scans could offer a significant advancement in predicting ER in postresection HCC patients.

## Figures and Tables

**Table 1:** Baseline characteristics of patients.

Variables	Non-ER (*n* = 59)	ER (*n* = 60)	*p*-Value	Training Group (*n* = 83)	Test Group (*n* = 36)	*p*-Value
Age(years), mean ± SD	62.68 ± 9.00	60.23 ± 12.18	0.431	61.88 ± 10.39	60.44 ± 11.62	0.506
Sex, *n* (%)			0.668			0.936
Male	49 (83.1)	48 (80.0)		67 (80.7)	30 (83.3)	
female	10 (16.9)	12 (20.0)		16 (19.3)	6 (16.7)	
AFP, *n* (%)			0.777			0.261
≤400 ng/mL	48 (81.4)	50 (83.3)		71 (85.5)	27 (75)	
>400 ng/mL	11 (18.6)	10 (16.6)		12 (14.5)	9 (25)	
Pathogenesis, *n* (%)			0.422			0.845
HBV	46 (78.0)	43 (71.7)		63 (75.9)	26 (72.2)	
HCV	2 (3.4)	6 (10.0)		5 (6.0)	3 (8.3)	
Other	11 (18.6)	11 (18.3)		15 (18.1)	7 (8.4)	
CNLC, *n* (%)			0.779			0.476
Ia	25 (42.4)	28 (46.7)		34 (41)	19 (52.8)	
Ib	18 (30.5)	19 (31.7)		27 (32.5)	10 (27.8)	
IIa	16 (27.1)	13 (21.7)		22 (26.5)	7 (19.4)	
BCLC, *n* (%)			0.973			0.476
0 + A	52 (88.1)	53 (88.3)		72 (86.7)	33 (91.7)	
B	7 (11.9)	7 (11.7)		11 (13.3)	3 (8.3)	
Pathologic MVI, *n* (%)			0.013			0.649
Absent	34 (57.6)	21 (35.0)		40 (48.2)	15 (41.7)	
Present	25 (42.4)	39 (65.0)		43 (51.8)	21 (58.3)	
Satellite nodules, *n* (%)			0.615			0.381
Absent	46 (78.0)	49 (81.7)		64 (77.1)	31 (86.1)	
Present	13 (22.0)	11 (18.3)		19 (22.9)	5 (13.9)	
Pathologic differentiation grade, *n* (%)			0.044			0.312
Low	8 (13.6)	18 (30.0)		15 (18.1)	11 (30.6)	
Median	37 (62.7)	35 (58.3)		53 (63.9)	19 (52.8)	
High	14 (23.7)	7 (11.7)		15 (18.1)	6 (16.7)	
Tumor size, cm	4.28 ± 2.52	5.71 ± 2.62	<0.001	5.09 ± 2.82	4.79 ± 2.27	0.571
Radiologic evidence of cirrhosis, *n* (%)			0.523			1.000
Absent	24 (40.7)	21 (35.0)		31 (37.3)	14 (38.9)	
Present	35 (59.32%)	39 (65.00%)		52 (62.7)	22 (61.1)	
CT wash-in, *n* (%)			0.714			0.600
Absent	3 (5.1)	4 (6.7)		6 (7.2)	1 (2.8)	
Present	56 (94.9)	56 (93.3)		77 (92.8)	35 (97.2)	
CT wash-out, *n* (%)			0.045			0.408
Absent	7 (11.9)	5 (8.3)		8 (9.6)	4 (11.1)	
Present	52 (88.1)	55 (91.7)		75 (90.4)	32 (88.9)	
CT measured infiltrative margin, *n* (%)			<0.001			1.000
Absent	41 (69.5)	20 (33.3)		43 (51.8)	18 (50)	
Present	18 (30.5)	40 (66.7)		40 (48.2)	18 (50)	
CT measured tumor number, *n* (%)			0.099			0.838
single	42 (71.2)	34 (56.7)		54 (65.1)	22 (61.1)	
multiple	17 (28.8)	26 (43.3)		29 (34.9)	14 (38.9)	
CT measured intratumoral necrosis, *n* (%)			0.637			0.851
Absent	34 (57.6)	32 (53.3)		47 (56.6)	19 (52.8)	
Present	25 (42.4)	28 (46.7)		36 (43.4)	17 (47.2)	
CT measured pseudocapsule, *n* (%)			0.397			0.708
Absent	36 (61.0)	32 (53.3)		46 (55.4)	22 (61.1)	
Present	23 (39.0)	28 (46.7)		37 (44.6)	14 (38.9)	
MRI wash-in, *n* (%)			0.978			1.000
Absent	5 (8.5)	5 (8.3)		7 (8.4)	3 (8.3)	
Present	54 (91.5)	55 (91.7)		76 (91.6)	33 (91.7)	
MRI wash-out, *n* (%)			0.978			1.000
Absent	5 (8.5)	5 (8.3)		5 (6.0)	5 (13.9)	
Present	54 (91.5)	55 (91.7)		78 (94)	31 (86.1)	
MRI measured infiltrative margin, *n* (%)			0.388			0.769
Absent	38 (64.4)	34 (56.7)		49 (59)	23 (63.9)	
Present	21 (35.6)	26 (43.3)		34 (41)	13 (36.1)	
MRI measured tumor number, *n* (%)			0.015			0.424
single	45 (76.3)	33 (55.0)		52 (62.7)	26 (72.2)	
multiple	14 (23.7)	27 (45.0)		31 (37.3)	10 (27.8)	
MRI measured intratumoral necrosis, *n* (%)			<0.001			1.000
Absent	45 (76.3)	27 (45.0)		50 (60.2)	22 (61.1)	
Present	14 (23.7)	33 (55.0)		33 (39.8)	14 (38.9)	
MRI measured pseudocapsule, *n* (%)			0.035			0.224
Absent	36 (61.0)	25 (41.7)		39 (47)	22 (61.1)	
Present	23 (39.0)	35 (58.3)		44 (53)	14 (38.9)	
LI_RADS, *n* (%)			0.402			0.444
3	4 (6.8)	4 (6.7)		4 (4.8)	4 (11.1)	
4	5 (8.5)	10 (16.7)		11 (13.3)	4 (11.1)	
5	50 (84.7)	46 (76.6)		68 (81.9)	28 (77.8)	
Radscore^CT^	0.38 ± 0.21	0.65 ± 0.25	<0.001	0.50 ± 0.27	0.55 ± 0.28	0.340
Radscore^MRI^	0.38 ± 0.24	0.68 ± 0.22	<0.001	0.51 ± 0.29	0.58 ± 0.24	0.201
Radscore^CT&MRI^	0.26 ± 0.23	0.76 ± 0.23	<0.001	0.49 ± 0.35	0.56 ± 0.31	0.343

ER—early recurrence.

**Table 2:** Multivariate logistic analysis for independent factors associated with early recurrence in HCC patients.

	CRP Model		Combined CT		Combined MRI		Combined CT and MRI	
	OR (95%CI)	*p*	OR (95%CI)	*p*	OR (95%CI)	*p*	OR (95%)	*p*
Tumor size	0.53 (0.26–1.10)	0.555	1.34 (1.09–1.64)	0.002 *	1.18 (0.96–1.45)	0.11	1.37 (1.04–1.80)	0.021 *
Pathologic differentiation grade	0.46 (0.23–0.92)	0.029 *	0.65 (0.30–1.42)	0.279	0.57 (0.27–1.20)	0.14	0.54 (0.20, 1.45)	0.221
MRI measured tumor number	3.91 (1.52–10.07)	0.005 *	NA	NA	2.24 (0.82–6.15)	0.12	3.20 (0.70, 14.52)	0.132
MRI measured intratumoral necrosis	2.74 (1.10–6.82)	0.031 *	NA	NA	3.13 (1.12–8.81)	0.03 *	4.60 (0.83, 25.54)	0.080
CT measured infiltrative margin	4.74 (1.91–11.77)	0.001 *	4.77 (1.74–13.09)	0.003 *	NA	NA	5.87 (1.25–27.44)	<0.024 *
CT wash-out	3.80 (1.07–13.51)	0.039 *	2.43 (0.57–10.33)	0.231	NA	NA	2.45 (0.36, 16.96)	0.363
Radscore^CT^	NA	NA	16.32 (9.19–138.84)	<0.001 *	NA	NA	NA	NA
Radscore^MRI^	NA	NA	NA	NA	23.27 (2.38–227.60)	<0.01 *	NA	NA
Radscore^CT&MRI^	NA	NA	NA	NA	NA	NA	30.98 (17.88–536.4)	<0.001 *

Note: CRP—clinical–radiological–pathological. * Factors showed statistical significance in multivariate logistic analysis.

**Table 3:** Comparison performance of CT, MRI, and CT and MRI models in the training and test cohorts with ROC.

	Training				Test					*p*	
	AUC (95%CI)	ACC	Sen	Spec	AUC (95%CI)	ACC	Sen	Spec		Training Set	Test Set
(1) Radiomics^CT^	0.820 (0.731, 0.909)	0.759	0.643	0.878	0.742 (0.552, 0.887)	0.778	0.722	0.833	1 vs. 3	0.001 *	0.032 *
(2) Radiomics^MRI^	0.833 (0.749, 0.916)	0.747	0.524	0.976	0.753 (0.559, 0.868)	0.750	0.833	0.667	2 vs. 3	0.001 *	0.039 *
(3) Radiomics^CT&MRI^	0.931 (0.879, 0.983)	0.867	0.809	0.927	0.909 (0.765, 0.957)	0.833	0.999	0.667	1 vs. 2	0.850	0.911
(a) Combined CT	0.894 (0.804, 0.948)	0.867	0.857	0.878	0.784 (0.585, 0.884)	0.750	0.778	0.722	a vs. c	0.044 *	0.010 *
(b) Combined MRI	0.856 (0.756, 0.922)	0.783	0.667	0.902	0.787 (0.612, 0.900)	0.750	0.833	0.667	b vs. c	0.001 *	0.024*
(c) Combined CT and MRI	0.955 (0.890, 0.981)	0.927	0.905	0.951	0.951 (0.792, 0.961)	0.916	0.990	0.833	a vs. b	0.464	0.983
									3 vs. c	0.083	0.434

AUC—area under the curve; ACC—accuracy; Sen—sensitivity; Spec—specificity; 95%CI—95% confidence interval. * Comparison of AUC between models showed statistical significance in the training set and test set, *p* < 0.05.

## Data Availability

As a matter of privacy, I do not wish to share my data, but they are available from the corresponding author upon reasonable request.
